# Feasibility of tissue-engineered tracheal and bronchial reconstruction using an in vivo bioreactor technique

**DOI:** 10.1016/j.xjtc.2026.102391

**Published:** 2026-04-16

**Authors:** Ziang Wang, Yuanyuan Xu, Jiazheng Huang, Ning Wang, Feng Mao, Zhongjie Wang, Qingquan Luo, Qiang Tan

**Affiliations:** Department of Thoracic Surgery, Shanghai Chest Hospital, Shanghai Jiao Tong University School of Medicine, Shanghai, China

**Keywords:** tissue-engineered airway, bronchial reconstruction, long-segment airway defects, tissue-engineered tracheal and bronchial reconstruction, in vivo bioreactor technique

## Abstract

**Objective:**

Reconstruction of long-segment airway defects remains challenging because previous attempts at tissue-engineered airway (TEA) replacement have failed to provide long-term follow-up data. We report the feasibility of a novel in vivo bioreactor technique for TEA reconstruction to correct long-segment tracheal and bronchial defects.

**Methods:**

The outcomes of 7 patients with end-stage tracheal lesions or proximal lung tumors requiring pneumonectomy who underwent radical lesion resection using a standard surgical technique, followed by TEA reconstruction with an in vivo bioreactor technique were retrospectively reviewed. An intramural nitinol stent was inserted between 2 layers of an acellular porcine dermal matrix, with 2 Port-a-Cath (Smiths Medical) catheters connected to a portable peristaltic pump. The TEA was continuously perfused with an antibiotic solution, and peripheral total nucleated cells were harvested and seeded into the implanted TEA substitute twice per week for 2 months.

**Results:**

The 7 patients (mean age, 49 years; range, 33-67 years; 4 men) underwent either tracheal (n = 5) or bronchial (n = 2) reconstruction. Two of the 7 patients died within 1 year due to fistula formation between the trachea and brachiocephalic artery. One other major morbidity event (upper anastomosis leakage) was resolved with a longer membrane stent. Three patients died due to tumor relapse (median follow-up, 2 years), whereas 2 patients are still alive (follow-up, 6.5 years). Two patients underwent biopsy-confirmed TEA re-epithelialization.

**Conclusions:**

The findings of this uncontrolled study suggest that TEA using an in vivo bioreactor technique is feasible for long-segment trachea and bronchial reconstruction. Further studies are needed to evaluate efficacy and safety.

**Figure:**
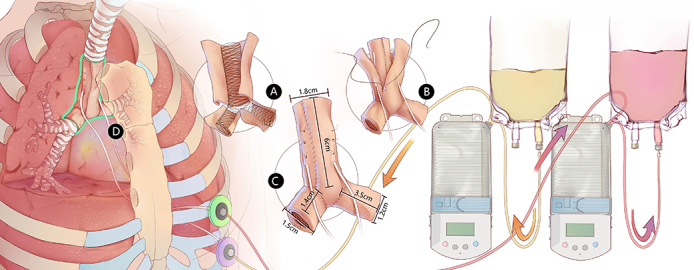
This novel approach ensured cell survival and promoted tissue regeneration in situ.

**Central Message**

The airway defect was reconstructed by tissue-engineered airway with the in vivo bioreactor technique.

**Perspective**

We report the feasibility of a novel in vivo bioreactor technique for TEA reconstruction to correct long-segment tracheal and bronchial defects. We believe our work will significantly contribute to the ongoing discussions regarding the future of tissue engineering and organ replacement.

The feasibility of tissue-engineered (TE) trachea replacement has been in doubt due to clinical application failure and academic misconduct.^1^ In animal experiments, more than 2 cm of the trachea were resected and immediately re-anastomosed in situ. This consummate TE trachea substitute, composed of autologous cells with normal tracheal structure, seemed to fail due to delayed revascularization. These results challenge the traditional tissue engineering technique characterized by a 3-dimensional (3D) cell culture on a biodegradable scaffold in vitro.^2^ The traditional tissue engineering technique involves two separate stages that include in vitro 3D cell-scaffold construct culture and in vivo regeneration of a TE substitute. However, it is extremely difficult to create a capillary network within the TE substitute in vitro because implanted TE substitutes can only obtain nutrition via tissue fluid infiltration and vessel ingrowth from surrounding recipient tissue.^3^ Unfortunately, the duration of this revascularization process is normally several months and during this time, most of the pre-seeded cells in the central part of the TE substitute die due to ischemia.^4^

To address this problem, an in vivo bioreactor composed of an implanted TE substitute perfused with intra-scaffold medium flow from 2 portable peristaltic pumps was introduced for in situ tissue regeneration.^5^, ^6^, ^7^ Pilot in vitro studies and animal experiments demonstrated 4 main advantages of this design. First, continuous medium perfusion maintained survival of the seeded cells. Second, the in vivo bioreactor functions to reseed the cells into the implanted TE substitute. Third, antibiotics were added to the perfusate to prevent infection. Fourth, various growth factors were added to the perfusate to maintain a regeneration niche within the implanted TE substitute. The concentration of the growth factors was easily adjusted in the perfusate to satisfy different regeneration stages.^8^

Based on these studies, the ethical committee of Shanghai Chest Hospital (Shanghai, China) approved clinic application of the proposed in vivo bioreactor technique during 2015. Here, the long-term follow-up results are presented.

## Methods

### Study Design and Oversight

The study was sponsored and conducted by Shanghai Chest Hospital, affiliated with Shanghai Jiaotong University. The protocol, written by Qiang Tan (principal investigator), focused on long-segment tracheal replacement in patients with tracheal defects unrepairable with traditional end-to-end anastomosis and bronchial replacement to avoid pneumonectomy in patients with extensive lung cancer. Institutional review board approval (KS1744) was granted November 23, 2017. Written informed consent for publication was obtained from all patients.

### Patients

Eligible patients underwent a standard preoperative evaluation and cardiopulmonary assessment. The study protocol was approved by the Ethics Committee of Shanghai Chest Hospital. A multidisciplinary team approved the inclusion and exclusion criteria. The inclusion criteria were long-segment tracheal defects (>6 cm) that could not be repaired by traditional end-to-end anastomosis due to excessive tension or proximal lung tumors invading the main bronchi where sleeve resection was not feasible and pneumonectomy would otherwise be required to achieve complete resection. The exclusion criteria were metastatic disease confirmed by positron emission tomography/computed tomography (CT) and brain magnetic resonance imaging, limited tracheal tumors that could be repaired by standard resection and direct end-to-end anastomosis, lung cancer requiring standard sleeve lobectomy, and inability to provide informed consent.

### Treatment

**Figure 1:**
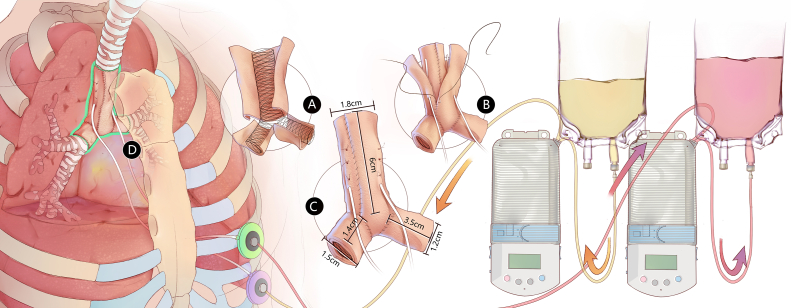
The airway defect was reconstructed by tissue-engineered airway with the in vivo bioreactor technique.

After enrollment in the study, a custom-made nitinol stent (Beijing Puyishengji Technology Co Ltd) was manufactured based on preoperative 3D CT imaging of the trachea. Radical resection of the airway lesion was performed using standard surgical techniques. The airway defect was reconstructed by TE airway (TEA) replacement with the in vivo bioreactor technique (Figure 1).

The TEA substitute constructed with the in vivo bioreactor technique was composed of a custom-made nitinol stent inserted between 2 layers of an acellular porcine dermal matrix (APDM) (Unitrump Biomedical Technology Co Ltd) with 2 Port-a-Cath catheters (Smiths Medical) embedded subcutaneously in the chest wall with the ends connected to 2 portable peristaltic pumps (ACE Medical Co Ltd) to maintain continuous medium perfusion within the TEA. One pump continuously delivered Ringer's solution with 100 μg/mL gentamicin into the TEA, while the other removed the waste. The speed of both pumps was adjusted to 10 mL/hour. On postoperative day 7, a blood sample (20 mL) was collected and peripheral total nucleated cells (TNCs) were isolated using the Res-Q 60 BMC system (Cesca Therapeutics Inc). The TNCs were injected directly into the implanted TEA via the embedded Port-a-Cath system. Ringer's perfusion was paused for 2 hours to allow for the TNCs to adhere to the scaffold and then resumed to remove the unadhered cells. TNC injections (0.9-1.8 × 10^8^cells) were performed twice a week (every Monday and Thursday) for 2 months.

For tracheal replacement, a membrane stent was placed postoperatively to cover the TEA and 2 anastomoses to prevent projection of the inner APDM layer into the airway to avoid obstruction and leakage of the bioreactor perfusate into the lungs, which could result in lethal pneumonia. For 2 bronchial replacement patients, stenting of the inner membrane was not necessary.

### Follow-up and Outcomes

Primary outcomes were 1-year mortality and morbidity related to the TEA procedure. Long-term survival and functional status were assessed as exploratory end points. Patients were followed up for 1 year. The outcomes of mortality and morbidity related to the TEA in vivo bioreactor design were assessed. Bronchoscopic examinations were performed weekly for the first month and every 3 months for 1 year. CT scans were performed every 3 months.

After 1 year, the patients were followed-up with to assess long-term mortality and morbidity outcomes. The median follow-up was 2 years (range, 2 months to 6.5 years), and the follow-up completion rate was 100% (all 7 patients completed follow-up with no loss to follow-up). Biopsy specimens were obtained from the left bronchial replacement of patient No. 1 at 3 months and from patient No. 4, who underwent tracheal replacement (6 cm) after inner membrane stent removal, at postoperative month 42. Pathological examinations confirmed regeneration of ciliated columnar epithelium covering the entire TEA inner surface.

### Data Collection

The last flow-up visit occurred on August 1, 2024. The following data collected for each patient included sex, age, medical history, type of operation, duration of hospitalization, 90-day mortality and morbidity, histopathological specimen examination, postoperative treatment, delayed complications (after 90 days), stent removal timing, and status at last follow-up visit.

## Results

### Patients

The study duration was from December 2015 to August 2024. The study cohort included 7 patients (4 men and 3 women; mean age, 49 years; age range, 33-67 years). The patient characteristics, types of preoperative treatment, and indications for inclusion are listed in Table 1.

**Table 1:** The patient characteristics, types of preoperative treatment, and indications for inclusion

Patient	Sex	Age (y)	Medical history	Type of airway disease	Description of localization	Type of preoperative treatment	Indication for study inclusion
1	M	56	Past smoker (30 pack-years), and COPD	Non–small cell lung cancer, squamous cell subtype	The opening of the left main bronchus is obstructed, with a mass protruding into the lumen	Docetaxel + cisplatin, 30 Gy radiotherapy and target therapy (erlotinib)	To avoid pneumonectomy
2	F	40	/	Tracheal adenoid cystic carcinoma	2 cm below the glottis, a cauliflower-like neoplasm is observed on the tracheal wall, extending 4.5 cm downward along the trachea	–	Extensive lesion not accessible to conventional surgery
3	F	33	/	Tracheal adenoid cystic carcinoma	A neoplasm in the upper segment of the trachea causes lumen narrowing, with the upper margin located 7 cm from the glottis and the lower margin 5 cm from the carina. The lesion length is approximately 5.5 cm	–	Extensive lesion not accessible to conventional surgery
4	M	49	Hypertension	Tracheal adenoid cystic carcinoma	Granulation tissue proliferation is observed in the mucosa of the tracheal terminus and carina. Tumor tissue, approximately 3 cm in length, is visible in the lower segment of the trachea at the level of the carina. The lumen of the right bronchus is patent, and the mucosa appears smooth	Conventional endoscopic	Extensive lesion not accessible to conventional surgery
5	M	48	/	Tracheal adenoid cystic carcinoma	Scattered nodular protrusions are seen in the upper segment of the trachea, 1.5 cm from the glottis. At 5 cm from the glottis, the tracheal mucosa appears uneven, with lumen narrowing over a length of approximately 6 cm, involving the carina and the openings of the left and right main bronchi.	Endoscopic treatment	Extensive lesion not accessible to conventional surgery
6	M	67	/	Non–small cell lung cancer, squamous cell subtype	The opening of the right main bronchus is obstructed, with a mass protruding into the lumen.	Palliative endoscopic treatment	To avoid pneumonectomy
7	F	54	/	Tracheal adenoid cystic carcinoma	A cauliflower-like neoplasm is observed in the right main bronchus, completely obstructing the lumen. The surrounding mucosa shows infiltrative tumor changes, involving approximately 1 cm of the membranous portion of the carina. In the right intermediate bronchus, between the 11 o'clock and 2 o'clock positions, the mucosa displays infiltrative tumor changes and hypertrophic proliferation, extending to the opening of the posterior segment of the right lower lobe	Endoscopic treatment	Extensive lesion not accessible to conventional surgery

*M*, Male; *COPD*, chronic obstructive pulmonary disease; *F*, female.

Two patients were included to avoid pneumonectomy due to non–small cell lung cancer. Three patients had extensive malignant tracheal lesions but previous treatment had failed. Two patients presented with malignant tumors extending to the distal trachea, carina, and both main bronchi.

### Procedures

**Figure 2:**
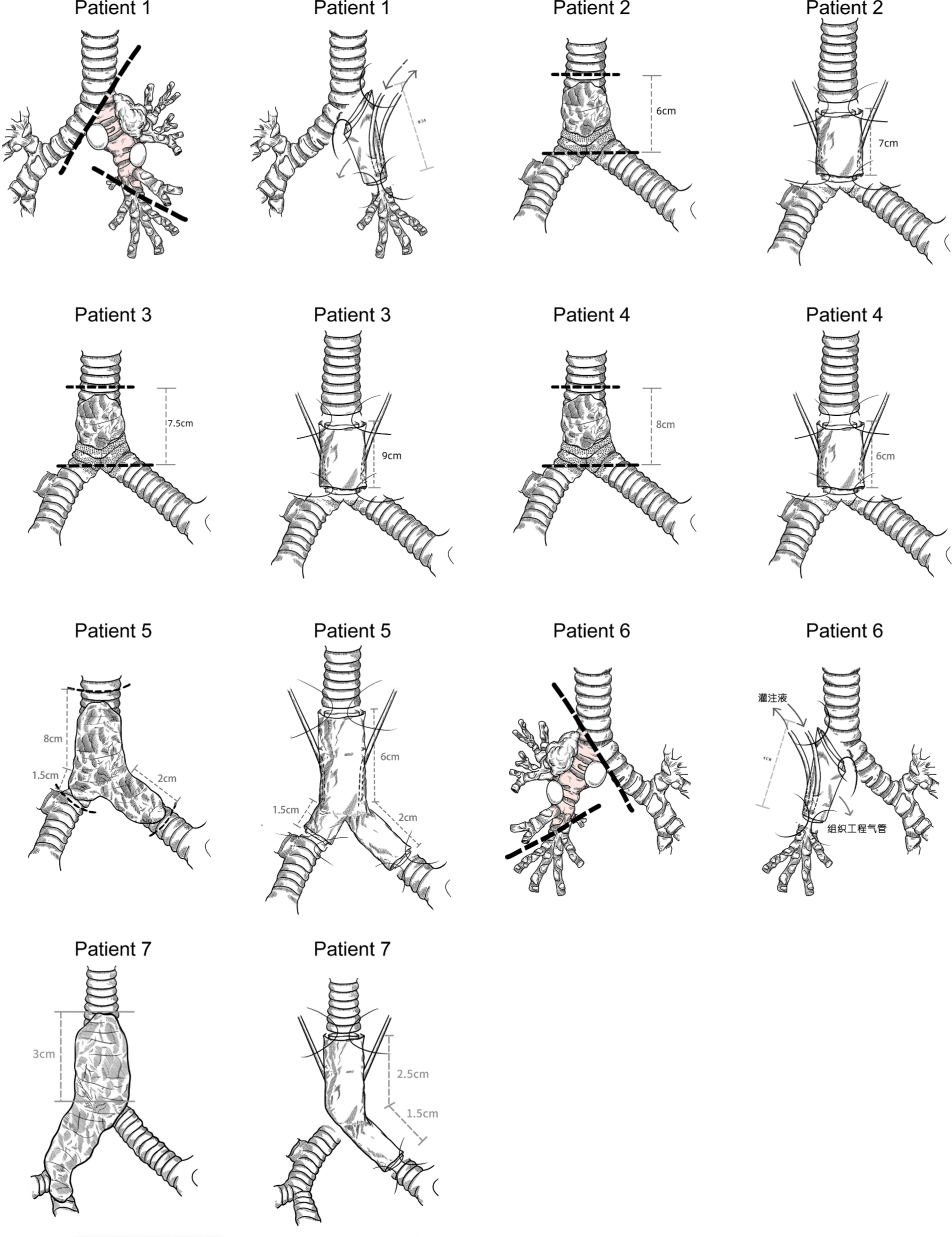
Illustrations of the intervention performed for each patient.

The interventions performed for each patient are detailed in Table 2 and Figure 2. TEA with the in vivo bioreactor design was applied for bronchial replacement in 2 patients; tracheal replacement in 3 patients; replacement of the trachea, carina, and main bronchus (*Y*-shape) in 1 patient; and replacement of the trachea, carina, and left main bronchus (*L*-shape) in 1 patient.

**Table 2:** Primary and secondary outcomes by type of intervention

Type of intervention	Date of operation	Patient no.	Hospital length of stay (d)	Postoperative survival (d)	Primary outcome: 1-y mortality	Secondary outcome: 1-y mortality	Cancer pathology	Type of postoperative cancer treatment
Tracheal transplantation Left upper lobe and LS6 segment resection	Feb 2015	1	35	388	Alive	No	pT3 N2 M0 Non–small cell lung cancer, squamous cell subtype	Radio chemotherapy Target therapy
Tracheal transplantation Trachea resection	Nov 2015	2	23	17	Die	Die	R1 resection Extended carcinoid tumor	NA
Tracheal transplantation Trachea resection	Nov 2015	3	17	31	Die	Die	R0 resection Extended carcinoid tumor	NA
Tracheal transplantation Trachea resection	Dec 2017	4	98	2510 (As of November 1, 2024)	Alive	Gastric retention Granuloma	R0 resection Extended carcinoid tumor	NA
Tracheal transplantation Trachea and carina resection	Mar 2018	5	87	1493	Alive	Gastric retention Granuloma	R0 resection Extended carcinoid tumor	NA
Tracheal transplantation Right upper and middle lobe resection	July 2018	6	13	746	Alive	Granuloma	pT4 N2 M0 Non–small cell lung cancer, squamous cell subtype	NONE
Tracheal transplantation Right pneumonectomy, distal tracheal, carina resection	June 2023	7	83	518 (As of November 1, 2024)	Alive	Anastomosis leakage Pneumonia	R0 resection Extended carcinoid tumor	NA

*NA*, Not available.

### Outcomes

The primary outcomes are detailed in Table 2. Among the 7 patients, 2 died within 1 year. Two patients died at the initial stage of the study due to fistula formation of the trachea and brachiocephalic artery caused by erosion of noncustom-made nitinol stents. Two patients who underwent bronchial replacement and 3 others who underwent tracheal replacement with custom-made nitinol stents had survived beyond 1 year (mortality rate, 0%).

The details regarding the secondary outcomes for each patient are provided in Table 2. The mean duration of hospitalization was 51 days (range, 13-98 days). Major 1-year morbidity events related to TEA occurred in 4 of the 7 patients, which included anastomosis leakage, gastric retention, pneumonia, and granuloma. For patient No. 7, who underwent right pneumonectomy with resection of the distal trachea and carina, an *L*-shaped TEA was used to connect the proximal trachea and left main bronchus. At postoperative month 1, the mediastinum had shifted totally to the right chest cavity. Leakage of the upper anastomosis was detected due to an increased shearing force. With venovenous extracorporeal membrane oxygenation, a longer *L*-shaped membrane stent was applied to re-cover the upper anastomosis fistula. The patient recovered and was eventually discharged but developed a granuloma of the left main bronchus anastomosis that required bronchoscopy every 2 months.

### Long-Term Follow-up

Two patients who underwent TEA bronchial replacement had died due to lung cancer relapse. Bronchoscopy confirmed full re-angiogenesis and re-epithelialization of the left main bronchus TEA substitute in patient No. 1, who received 2-regimen chemotherapy (docetaxol + cisplatin), 30-Gy radiotherapy, and targeted therapy (erlotinib). To the best of our knowledge, this is the first report of a TEA patient who underwent tumor adjuvant therapy and achieved complete bronchus regeneration without foreign body replacement.

According to the literature, previous attempts at bioengineered carinal reconstruction failed within 90 days due to delayed revascularization and recurrent infection. For patient No. 6, an 8-cm tracheal segment was resected together with the carina, entire right main bronchus, and a 3-cm segment of the left main bronchus. The long-segment airway defect was reconstructed using a *Y*-shaped TEA. The patient survived for 4.5 years and died from adenoid cystic carcinoma relapse. She was under quarantine during the COVID-19 pandemic and failed to appear for follow-up examinations over the past 2 years. Through a follow-up interview by telephone, she described usual activities without dyspnea, which is indicative of airway obstruction. To the best of our knowledge, she was the first patient to achieve long-term survival after *Y*-shaped TEA replacement.

Carinal resection with sleeve pneumonectomy is a challenging procedure with relatively high mortality and morbidity rates. In a previous report of stented aortic matrices or autologous substitute for carinal reconstruction, all patients had died within 90 days.^9^ Patient No. 7 was the first to undergo carinal resection with sleeve pneumonectomy with the TEA replacement procedure who survived for more than 1 year. She developed a granuloma that occluded the lower left main bronchus anastomosis that required bronchoscopy every 2 months.

Patient No. 4, who was also diagnosed with adenoid cystic carcinoma, underwent tracheal resection of 8 cm, which was repaired with a 6-cm TEA replacement. The patient returned to normal life and the implanted membrane stent was withdrawn by bronchoscopy at a 3-year follow-up. The implanted APDM was totally biodegraded and replaced by regenerated granulation tissue. A biopsy at the 4-year bronchoscopy follow-up confirmed regeneration of ciliated columnar epithelial cells of the entire TEA substitute. A bronchoscopy video is available in Video 1. To the best of our knowledge, this patient is the first to survive for more than 5 years after TEA replacement with detailed follow-up data.

## Discussion

Belsey mentioned in the 1960s, “The successful solution of the problem of trachea reconstruction may mark the end of the expansionist epoch in the development of surgery.”^10^ More than a half century later, long-segment airway replacement remains clinically challenging due to 2 seemingly insurmountable biological obstacles: delayed revascularization and infection.

Tracheal blood vessels are fragile and enter the trachea through lateral tissue pedicles in a segmental fashion and, therefore, unsuitable for direct vessel anastomosis revascularization as performed with transplantation of other organs. With the use of animal experiments, more than 2 cm of the trachea was successfully resected and immediately reanastomosed in situ. This consummate trachea substitute, composed of autologous cells with a normal tracheal structure, seemed to fail due to delayed revascularization, thereby highlighting complications of tracheal transplantation and TEA substitution.^11^ Tracheal allografts heterotopically wrapped in omentum and autografts with vascularized fascial flaps have been clinically tested to restore the blood supply.^12^^,^^13^ However, all indirect revascularization techniques are tedious with inconsistent results. Moreover, the trachea opens to the air, which is full of bacteria that proliferate much faster than the normal cells in airway substitutes. Notably, bacterial infection disrupts normal tissue regeneration and can cause lethal pneumonia, thereby frustrating research of all kinds of artificial substitutes, which have only been successful in sterile situations, such as bone and vessel replacement.^14^

To overcome these challenges, we pioneered an in vivo bioreactor technique using an implanted TE substitute perfused with an intrascaffold flow of medium created by an extracorporeal portable pump system for in situ tissue and organ regeneration. There are 4 main advantages to the TEA bioreactor technique. First, continuous medium perfusion enhanced survival of the seeded cells both within and on the surface of the scaffold, which is especially important for regeneration of the tracheal and bronchial epithelium. Second, the bioreactor can function as a cell re-seeding system. In the clinic, most airway obstructions require emergency surgery because patients cannot wait months for reconstruction of a TEA in the lab. In extreme cases, a scaffold could be implanted first before harvesting the appropriate tissue. After the initial procedure, the cells were isolated and expanded in vitro, then seeded back through the in vivo bioreactor as a daily infusion. In this study, TNCs, a mixture of stem cells and immunocytes, were used mainly because in vitro expanded stem cells have not yet been approved for clinical use in China. Various angiogenesis growth factors were found in the collected perfusate waste. Because Ringer's solution with gentamicin was used without any growth factor supplements as an inlet perfusate, these angiogenesis growth factors were mainly secreted by the re-seeded cells in the ischemic niche of the implanted TE tracheal substitute. The secretion of growth factors is circumstantial evidence of the contribution of reseeded cells to regeneration of the TEA. Third, various growth factors could be added to the perfusate as a growth factor cocktail, which is the easiest and most efficient method to control the concentration of functional growth factors within the implanted TEA. Lastly, the addition of antibiotics to the perfusate effectively controlled infection within the TEA.

Here, we present long-term survival data for TEA implantation with an in vivo bioreactor technique. Two of the 7 patients died within 1 year. At the initial stage of this study, a clinically available nitinol stent was chosen, which was tougher and larger than the natural trachea. These noncustom-made stents are usually temporarily applied to push aside the tracheal tumor to immediately relieve airway obstruction. In the scenario of TEA, the nitinol stents were covered only by 2 thin layers of biodegradable APDM. However, catastrophic fistulas of the trachea and brachiocephalic artery formed within 2 months. Following approval by the ethics review board, we modified the protocol to switch to custom-made nitinol stents with sparse densities and smaller diameters, which prevented fistula formation of the brachiocephalic artery in the following cases.

Two patients underwent TEA biopsy during follow-up. Serial bronchoscopy follow-up examinations confirmed gradual biodegradation of the APDM and granulated tissue ingrowth. For patients Nos. 1 and 4, the APDM had fully biodegraded and the airway had regenerated with no evidence of cartilage tissue regeneration. In our opinion, re-angiogenesis and re-epithelialization are imperative for airway regeneration, whereas cartilage tissue can be readily replaced with a nitinol stent. Because the airway was regenerated, the multidisciplinary team selected adjuvant lung cancer therapy for patient No. 1 and removal of the implanted membrane stent for patient No. 5 due to the absence of granulation overgrowth.

Many previous highly publicized reports of TEA replacement were later retracted due to academic misconduct. The current series provides some of the longest documented survival times with bronchoscopic and histologic evidence of re-epithelialization.

### Limitations

This was a retrospective study with a limited number of patients. Although TEA with the in vivo bioreactor technique is a promising method to promote revascularization while avoiding infection problems that occur during long-segment airway replacement, prospective clinical trials are required to confirm these advantages.

## Conclusions

In this retrospective study, the clinic application of TEA with the in vivo bioreactor technique demonstrated feasibility for long-segment tracheal and bronchial reconstruction in patients with complex airway defects. The study showed that, despite the initial catastrophic complications with noncustom-made stents, the in vivo bioreactor technique led to promising outcomes, including successful re-epithelialization and improved patient survival.

However, the study was limited by the small sample size, which prevented definitive conclusions regarding the efficacy and safety of the in vivo bioreactor technique. Hence, further research is essential, including larger, prospective clinical trials, to confirm these findings and to refine the technique. Future studies should also explore the potential for broader applications of this technology in other TE organ regeneration, such as TE esophagus and bone.

In summary, although promising, the in vivo bioreactor technique for TEA reconstruction requires further validation to establish feasibility in clinical practice. Continued innovation and rigorous evaluation will be critical to overcome the challenges associated with airway reconstruction and improve patient outcomes.

## Conflict of Interest Statement

The authors reported no conflicts of interest.

The *Journal* policy requires editors and reviewers to disclose conflicts of interest and to decline handling or reviewing manuscripts for which they may have a conflict of interest. The editors and reviewers of this article have no conflicts of interest.

## Supplementary Data

Video 1 Bronchoscopic follow-up of patient no. 4 at 4 years after tracheal resection and tissue-engineered airway (TEA) reconstruction, showing complete biodegradation of the APDM scaffold and confirmed ciliated epithelial regeneration. Video available at: https://www.jtcvs.org/article/S2666-2507(26)00198-7/fulltext.
